# Dietary Supplement of *Amomum villosum Lour.* Polysaccharide Attenuates Ulcerative Colitis in BALB/c Mice

**DOI:** 10.3390/foods11223737

**Published:** 2022-11-21

**Authors:** Donghui Luo, Jiao Zeng, Jingjing Guan, Yuanyuan Xu, Rui-Bo Jia, Jin Chen, Guili Jiang, Chunxia Zhou

**Affiliations:** 1 School of Food Science and Engineering, Guangdong Ocean University, Yangjiang 529500, China; 2 Chaozhou Branch of Chemistry and Chemical Engineering Guangdong Laboratory, Chaozhou 521000, China

**Keywords:** *Amomum villosum Lour.* polysaccharide, ulcerative colitis, gut flora

## Abstract

*Amomum villosum Lour.* (*A. villosum*), a comestible medicinal plant, has been traditionally used in China to treat diarrhea, stomach fullness, and abdominal distension. Polysaccharide, the main chemical component of *A. villosum*, has been shown to possess potential antioxidant and glycosidase inhibitory activities; however, whether it has anticolitis activity is unknown. The aim of this research was to evaluate the anticolitis effects of *A. villosum* polysaccharide (AVLP) in BALB/c mice. The results showed that AVLP administration significantly reversed body weight loss, colon shortening and colon weight gain and decreased the levels of proinflammatory cytokines and chemokines in colitis mice (*p* < 0.05). AVLP administration also maintained intestinal barrier function by the upregulation of ZO-1 protein expression (*p* < 0.05). In addition, high-throughput sequencing analysis showed that AVLP possessed a great regulatory effect on the growth of *Adlercreutzia*, *Clostridium*, *Streptococcus*, *Parabacteroides*, *Helicobacter*, *Odoribacter,* and *Alistipes* (*p* < 0.05, LDA score > 2). The correlation analysis revealed that the protective effects against colitis of AVLP were highly correlated with intestinal bacterium regulation. These results suggest that AVLP intake could serve as a prospective nutritional strategy for inflammatory bowel diseases.

## 1. Introduction

Ulcerative colitis (UC), one of the representative inflammatory bowel diseases (IBD), is usually characterized by bloody stool, diarrhea and intestinal mucosal injury [1]. The occurrence and prevalence of UC have continuously increased worldwide, particularly in developing countries [2]. Common treatment strategies for UC include antiphlogistic and immune-suppressive agents, such as corticosteroids, antibiotics and 5-aminosalicylic acid [3]. Nevertheless, the side effects and high costs of drug treatment remain to be addressed [4,5].

Recently, polysaccharides have attracted wide attention from researchers due to their safety, low cost, and multitarget therapeutic efficacy [6]. The accumulating reports have adequately demonstrated that some natural polysaccharides possess hypoglycemic activity, immunomodulatory activity, and the ability to regulate intestinal flora [7,8,9]. In addition, some scientists have also found that dietary polysaccharides could treat UC safely and effectively. For instance, the *Rauvolfia verticillata (Lour.) Baill*. polysaccharides have been shown to alleviate UC by adjusting NF-κB signal transduction pathways, balancing the composition of intestinal flora, and repairing intestinal mucosa [10]. Guar gum showed latent beneficial effects on UC, attributed to the proliferation of probiotic bacteria [11]. Rhamnogalacturonan possesses certain positive effects on UC by inhibiting inflammation, protecting intestinal epithelial cells, and reshaping the intestinal flora [6]. *Dendrobium* polysaccharides were found to ameliorate UC through inhibiting NLRP3 inflammasome and downregulating the *β*-arrestin1 signaling pathway [12]. *Dandelion* polysaccharides improved UC by regulating the IL-6/STAT3 pathway [13]. To sum up, polysaccharide is a promising resource for the treatment or adjuvant treatment of UC.

*Amomum villosum Lour.* (*A. villosum*), a perennial herb of dual-purpose for medicine and food, is widely distributed in the Fujian, Guangdong, Guangxi, and Hainan provinces of China [14]. The increasing data recorded show that *A. villosum* possesses multiplicate bioactivities, such as antioxidant, antitumor, anti-inflammatory, and bacteriostasis activity [15,16]. Polysaccharides are the main chemical constituents of *A. villosum*. However, the research on *A. villosum* has mainly focused on the volatile oil [17]. Previously, we obtained an undigestible *A. villosum* polysaccharide (AVLP) with a high extraction rate (about 15%). In the current research, our goal is to evaluate the anticolitis activity and mechanisms behind potential therapeutic effects of AVLP in BALB/c mice.

## 2. Materials and Methods

### 2.1. Materials and Reagents

The dried *A. villosum* was provided by Zhonghua Chunsharen Professional Cooperative in Yangchun City (Guangdong Province, China) and stored at room temperature for further use.

The series of chemical reagents and standards, including multiple monosaccharide standards and dextran sulfate sodium salt (DSS), were purchased from Sigma-Aldrich (St. Louis, MI, USA). Interleukin (IL)-6 and TNF-α immunosorbent kits were obtained from Nanjing Jiancheng Biological Engineering Institute (Jiangsu Province, China). Commercial kits associated with RT-PCR were obtained from Takara Bio (Kusatsu, Japan). Other chemical reagents used in the current research were of analytical grade and were purchased from Guangzhou Chemical Reagent Factory.

### 2.2. Extraction of AVLP

AVLP was extracted via ultrasound-assisted enzymatic extraction. Briefly, the raw material of *A. villosum* was pretreated for 60 min with a double enzyme (1% pectinase + 1% cellulase, pH 4.5, 50 °C). Subsequently, the mixture was treated with ultrasound at a power of 400 W for 30 min and was then bathed in water (90 °C) for 2 h. After cooling to room temperature, the supernatant was obtained via centrifugation (2000× *g*, 20 min), followed by intercept processing with an ultrafiltration membrane (3 kDa cut-off) five times. The concentrate was dissolved and precipitated with 80% ethanol (*v*/*v*) by maintaining it at 4 °C for 24 h. After centrifugation, the precipitate was collected, and the protein was removed. Finally, the AVLP was obtained by concentrating and lyophilizing.

### 2.3. Monosaccharide Composition Analysis

The composition of monosaccharide was analyzed by referring to a method in previous reports [18]. Briefly, the AVLP powder (10 mg) was hydrolyzed using 4 mol/L trifluoroacetic acid at 110 °C for 8 h, and the superfluous acid was removed by adding methanol. Then the hydrolysates were derivatized with 1-phenyl-3-methyl-5-pyrazolone (PMP) (70 °C, 100 min), while, the excess PMP was removed by trichloromethane. Finally, the sugar derivatives were filtered through a 0.22 μm aqueous phase filter and injected into the Agilent ZORBAX Eclipse XDB-C18 column (5 μm × 4.6 mm × 150 mm). The wavelength was 250 nm.

### 2.4. Molecular Weight Analysis

The molecular weight of AVLP was analyzed via a method from previous reports [19]. Briefly, the AVLP sample was eluted with 0.1 mol/L NaNO_3_ solution to 5 mg/mL, then the solution was percolated using a 0.45 μm filter and injected into the chromatographic columns (Ultruhydrogel 2000 and 1000) linked in series. The column temperature was 30 °C, the flow rate was 0.5 mL/min, and the dn/dc was 0.138 mL/g. The raw data were analyzed using ASTRA 6.1 software (Wyatt Technology, Santa Barbara, CA, USA).

### 2.5. FT-IR Spectrometry

The AVLP powder (2 mg) and KBr powder (40 mg) were mixed and squashed into slices. The spectra were recorded using a Bruker VERTEX 33 spectrometer (Bruker Corporation, Ettlingen, Germany) within the range of 4000 to 400 cm ^−1^ [20].

### 2.6. Animal Experiment

BALB/c mice (46 mice, 20 ± 3 g) were purchased from the Experimental Animal Center of Sichuan University (Sichuan province, China). All animals were housed in a stable, sterile environment (24–26 °C, 50–60% relative humidity) with a 12 h light/12 h dark cycle and free access to water and food. The animal experiment was carried out after 7 days of adaptive feeding. All animals were fed with standard diets (containing 10% kcal fat, 20% kcal protein, and 70% kcal carbohydrate) during the experiment. All procedures related to animals in this study were approved by the School of Food Science and Engineering of Guangdong Ocean University (No: 2021-036).

The dose of AVLP was determined according to the conversion of the human clinical dosage and the body surface area of mice, as well as the daily consumption of *A. villosum* (10 g/d for 60 kg human model) recommended by herbalist doctors and the approximate extraction yield (15%) of AVLP. The mice were fed with the same dose as the clinical equivalent, and the human-to-mouse conversion coefficient was 9.1. Thus, the total dose for a mouse for one day was (10 g/60 kg) × 9.1 × 15% ≈ 200 mg (i.e., 200 mg/kg/day for mice). In addition, a slightly higher dose (400 mg/kg/day) was selected to further investigate the anticolitis activity of AVLP.

All animals were randomly split into four groups: one group was used as a control (healthy group, 10 animals) and the other three groups were DSS-induced colitis groups. The colitis animal model was induced by substituting the potable water with a 3.5% DSS solution. The specific distribution was as follows: (1) Ctrl group (10 animals); (2) DSS group (12 animals); (3) DSS group supplemented with 200 mg/kg for 14 days (AVLP200 group, 12 animals); and (4) DSS group supplemented with 400 mg/kg for 14 days (AVLP400 group, 12 animals). When the experiment was over, all animals maintained fasting for 12 h, and the whole blood was collected under anesthesia. The colon was collected after sacrifice, and the colon length and colon weight (5 cm) of all animals were measured and recorded. Part of the colon was placed in 4% paraformaldehyde solution for histopathological and immunoblot analysis, while the remaining colon samples were stored in an ultra-low-temperature refrigerator (−80 °C) for RT-qPCR analysis and biochemical analysis. Animal feces were gathered and placed in liquid nitrogen for gut flora sequencing.

### 2.7. Serum Parameter Analysis

The serum TNF-α and IL-6 levels were detected using kits, referring to the instructions.

### 2.8. Histopathology Analysis

The colon fixed with 4% paraformaldehyde solution was embedded in paraffin and stained with hematoxylin and eosin (H&E), referring to a previous method [21]. The pathological features of stained sections (4 μm thick) were observed via light microscopy (×400).

### 2.9. Immunohistochemistry Analysis

The analysis of ZO-1 protein expression was performed by referring to a previous report [22]. The fixed colon (4 μm thick) was washed four times with PBS solution, followed by soaking treatment for 15 min in hydrogen peroxide (3%). The section was maintained with primary antibody for 12 h at 4 °C after blocking for 20 min. Afterward, the section was washed with PBS four times and incubated with secondary antibody for 60 min, then coupled with horseradish peroxidase for 30 min at room temperature. Tissues were rinsed with PBS three times before visualizing. Finally, the sections were dehydrated in ethanol, cleared in xylene, and mounted with Permount TM Mounting Medium. The colon tissue sections were observed via light microscopy, and the H-Score was calculated according to the following formula [23,24]:

H-SCORE = (percentage of weak intensity × 1) + (percentage of moderate intensity × 2) + (percentage of strong intensity × 3)

### 2.10. Quantitative Reverse Transcription PCR Analysis

The total RNA of colon tissues was extracted using a kit (Takara, Japan), and the cDNA was synthesized using a reverse transcription kit (Takara, Japan). PCR was carried out based on a CFX96 Real-Time PCR System (Bio-Rad, Hercules, CA, USA). The primer sequences are listed in Table 1, and the expression level was calculated via the 2^−ΔΔCt^ formula after normalization to GAPDH.

### 2.11. Intestinal Flora Analysis

The genomic DNA of enteric bacteria was obtained and assayed, by referring to previous methods [25]. The primers of 16S rRNA gene amplification were 338F (5′-CCTACGGRRBGCASCAGKVRVGAAT-3′) and 806R (5′-GGACTACNVGGGTWTCTAATCC-3′). The analysis of gut flora was carried out by Suzhou Panomico Biomedical Technology Co., Ltd. (Suzhou, China).

The overall bacterial composition at the OUT level was showed by partial least squares discriminant analysis (PLS-DA) and hierarchical clustering tree analysis using SIMACA 14.1 software. The divergence between groups was determined at the phylum and genus levels. The correlation between the microorganism at the genus level and an improvement in colitis was determined using R software (Ver. 3.3.3) and Cytoscape (Ver. 3.6.0).

### 2.12. Statistical Analysis

The statistical analysis was carried out based on a one-way analysis of variance (ANOVA) followed by Duncan’s test using SPSS 22 software. All results are expressed as the mean ± standard error. *p* < 0.05 was judged as indicating a significant difference.

## 3. Results and Discussion

### 3.1. Characterization of AVLP

**Figure 1:**
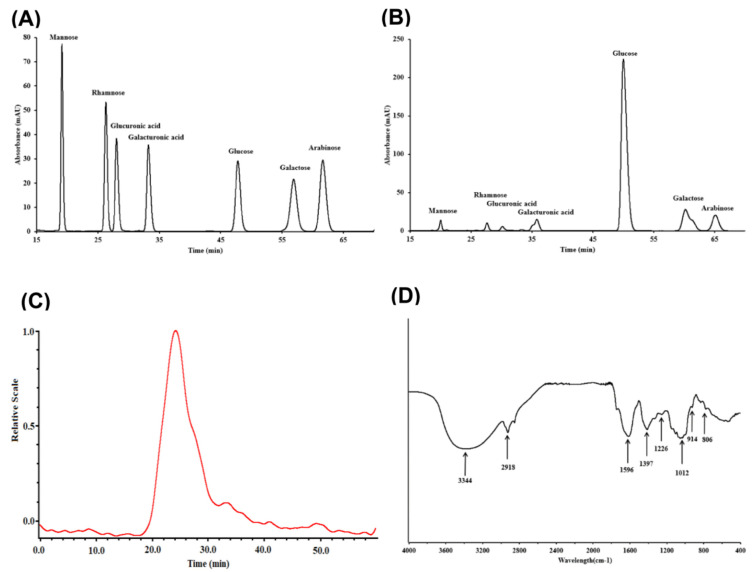
Characterization of AVLP. (**A**) HPLC chromatogram of a mixed solution of standard monosaccharides; (**B**) HPLC chromatogram of AVLP; (**C**) Molecular weight distribution of AVLP; (**D**) FT-IR spectrum of AVLP.

The essential information of AVLP is shown in Figure 1, containing the monosaccharide composition, molecular weight, and FT-IR spectra. Figure 1A,B indicate that the AVLP was composed of mannose, rhamnose, glucuronic acid, glucose, galactose, xylose, and arabinose. Among them, glucose was the major monosaccharide with a molar proportion of 89.40%. The average molecular weight of AVLP was 416.3 kDa (Figure 1C). The FT-IR spectrum is shown in Figure 1D, the absorption of -OH and C-H in AVLP appeared at 3344 cm^−1^ and 2918 cm^−1^, respectively. The peaks at 1397 cm^−1^ and 1596 cm^−1^ represent the C=O of acetyl or carboxylate groups. The peak at 1226 cm^−1^ corresponds to O=S=O. The absorption bands at 1012 cm^−1^ are due to the C-O-C stretching vibration. The absorption at 806 cm^−1^ is the band of C-O-S tensile vibration.

### 3.2. Effects of AVLP on Pathological Changes in Colitis Mice

*A. villosum*, a traditional Chinese medicine, is used to treat gastrointestinal diseases [26]. However, there are few modern *A. villosum* medicines or functional foods on the market, mainly due to the lack of in-depth and systematic studies on the relationship between its chemical composition and pharmacological activity. In the current study, we aimed to provide experimental evidence to support AVLP as a functional ingredient for alleviating colitis.

**Figure 2:**
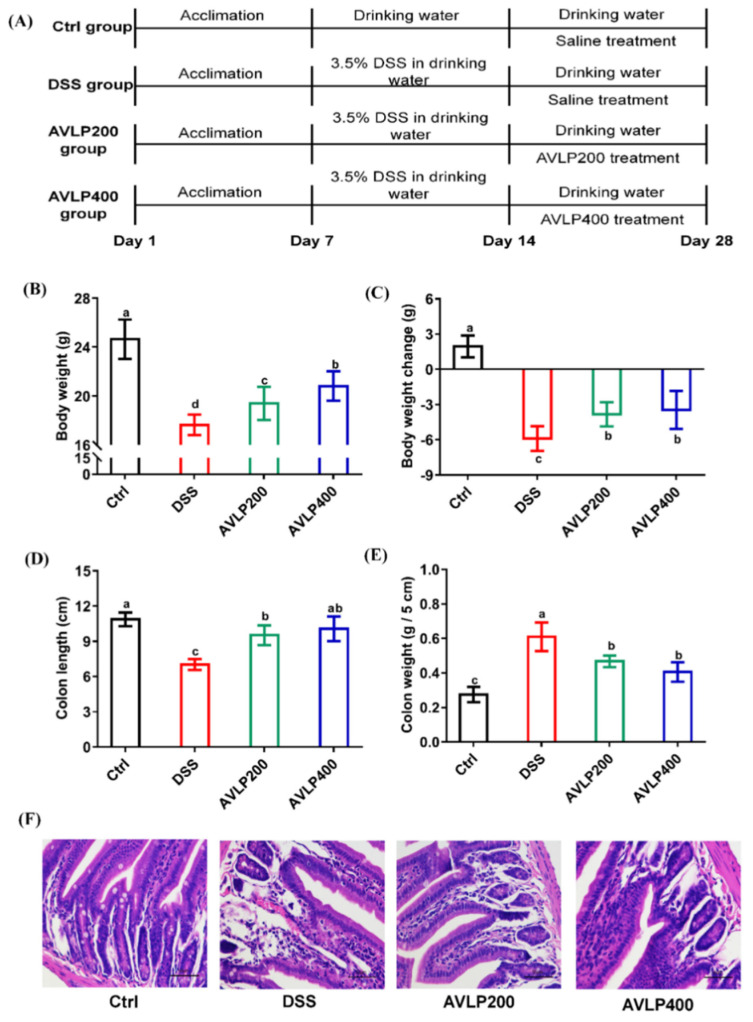
Effects of AVLP on pathological symptoms in colitis mice. (**A**) Schematic diagram of experimental design. (**B**) Body weight. (**C**) Body weight change (14–28th day). (**D**) Colon length. (**E**) Colon wet weight (5 cm). (**F**) Representative images of colon sections. Significant differences (*p* < 0.05) are indicated with different letters.

The animal model was established as exhibited in Figure 2A. Figure 2B,C display the effects of AVLP administration on the body weight of colitis mice. Compared with the Ctrl group, the body weight of the DSS treatment group was significantly reduced (*p* < 0.05), with a weight loss of 24.66%. Nevertheless, the animals in the AVLP administration groups (AVLP200 and AVLP400 groups) were heavier than those in the DSS group (*p* < 0.05). Furthermore, the colon length and wet weight are sensitive indicators of colitis severity. Our results suggest that AVLP treatment also significantly increased the colon length (Figure 2D), with change rates of 35.75% (AVLP200 group) and 43.31% (AVLP400 group) (*p* < 0.05), and significantly lightened the colon wet weight (Figure 2E), with change rates of 22.95% (AVLP200 group) and 32.77% (AVLP400 group) (*p* < 0.05). In addition, pathological observations are shown in Figure 2F. Compared with the Ctrl group, the obvious goblet cell lesions of the colon in the DSS group were reduced, the epithelial layer and crypt structure were damaged, and inflammatory cells were widely infiltrated, which are typical pathological features of UC development. Nevertheless, AVLP intervention significantly alleviated the damage to colon tissue. The above results show that AVLP administration possesses potential positive effects on colitis in mice.

### 3.3. Effects of AVLP on Inflammatory Cytokines and Chemokines in Colitis Mice

**Figure 3:**
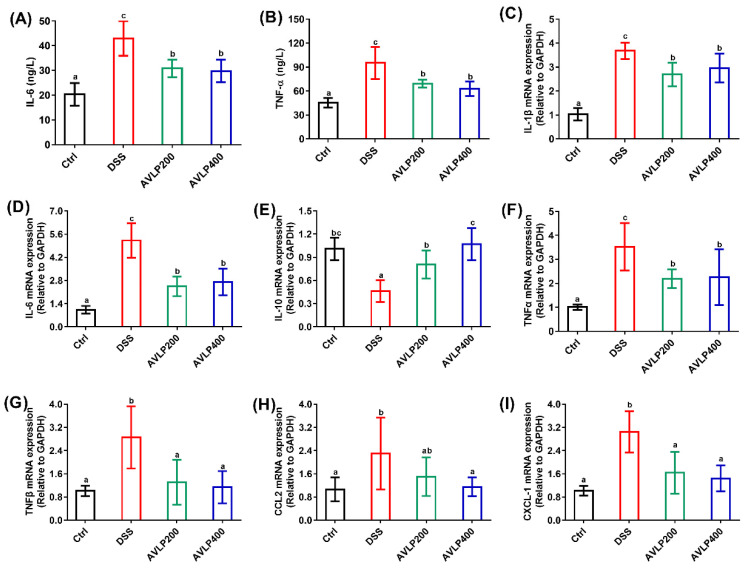
Effects of AVLP on inflammatory cytokines and chemokines in colitis mice. (**A**) Serum IL-6. (**B**) Serum TNF*-α*. (**C**) IL-1*β* mRNA expression. (**D**) IL-6 mRNA expression. (**E**) IL-10 mRNA expression, (**F**) TNF*α* mRNA expression, (**G**) TNF*β* mRNA expression, (**H**) CCL2 mRNA expression, (**I**) CXCL-1 mRNA expression. Significant differences (*p* < 0.05) are indicated with different letters.

Some cytokines, including IL-6 and TNF-*α*, are relevant to multiple physiological and immune processes. An increase in their serum concentrations is also an important biomarker for judging the development of colitis [27,28]. Furthermore, IL-6 and TNF-*α* have emerged as promising anti-inflammatory targets for the treatment of IBD [29]. Active substances that could reduce the serum levels of inflammatory factors may possess physiological activity to improve colitis [30]. As displayed in Figure 3A,B, the results showed that the levels of TNF-*α* (95.25 ± 20.06 ng/L) and IL-6 (42.96 ± 7.01 ng/L) in the DSS group were significantly elevated compared with those in mice from the Ctrl group (*p* < 0.05), and the increase rates were approximately 110.86% and 111.26%, respectively. However, AVLP treatment (both AVLP200 and AVLP400) prominently decreased the concentrations of IL-6 and TNF-*α* in serum (*p* < 0.05). In comparison to the DSS group, the decrease rates in the AVLP200 group were 31.97% (TNF-*α*) and 29.07% (IL-6), and the decrease rates in the AVLP400 group were 34.08% (TNF-*α*) and 30.66% (IL-6). There was no significant difference in the serum concentrations of inflammatory factors between the AVLP200 and AVLP400 groups (*p* > 0.05). These results demonstrate that AVLP administration decreased inflammatory reactions in colitis mice.

When the body is in a state of inflammatory disease, the colon immune cells are activated, which boosts the secretion of cell factors to elevate neutrophil infiltration, increase intestinal mucosa injury, and drive some macroscopic pathological symptoms of UC [31]. Increasing numbers of studies have demonstrated that the gene expression levels of pro-inflammatory cytokines and chemokines are elevated in sufferers of inflammatory bowel disease and in experimental colitis model animals induced by DSS [32]. As a consequence, we also assessed the effect of AVLP administration on enteric immune responses by measuring the mRNA relative expression. The results are shown in Figure 3C–I. The relative expression levels of some genes, involving IL-1*β*, IL-6, TNF-*α*, TNF-*β*, CCL2 and CXCL-1, were significantly elevated, while the IL-10 expression in the DSS group was prominently lowered in comparison to that in the Ctrl group (*p* < 0.05), suggesting that DSS induced a severe inflammatory reaction and intestinal barrier dysfunction. However, in comparison to those in the DSS group, the IL-1*β*, IL-6, TNF-*α*, TNF*-β* and CXCL-1 expression levels were significantly decreased and IL-10 relative expression was significantly upregulated in the AVLP200 and AVLP400 groups (*p* < 0.05). Furthermore, the CCL2 expression level in the AVLP400 group was also significantly downregulated (*p* < 0.05). Upregulated TNF*-a* and TNF*-β* could give rise to enteric mucosal damage by activating the caspase-dependent signal pathway and influencing the NF-κB signal pathway [33]. In vitro studies of intestinal segments excised from patients with IBD showed a remarkable increase in IL-1*β* expression [34]. The similar damaging effect of IL-1*β* on intestinal mucosal cells may work in combination with other cytokines to cause mucosal damage in IBD [35]. Furthermore, TNF-*α* and IL-l*β* are potent inducers of IL-6 [36]. Increased IL-6 mRNA expression may be associated with the upregulation of STAT3 signaling, accelerating the conversion of CD4+T cells into Th17 cells [35]. The activation of proinflammatory chemokines such as CCL2 and CXCL-1 is also responsible for the aggravation of colitis, attributed to adjustments to the transportation of leukocytes [37]. IL-10 secreted by T cell subsets and innate cells could reduce antigen presentation by restraining MHC II expression and T lymphocyte activity and inhibiting inflammatory cell production. Moreover, IL-10 could also reduce inflammation through restraining the production of inflammatory cytokines [38]. These findings show that the positive effects of AVLP on experimental colitis may be due to its anti-inflammatory activity by restraining the genetic expression of inflammatory cytokines and chemokines and by upregulating IL-10 expression.

### 3.4. Effect of AVLP on ZO-1 Protein Expression in Colitis Mice

**Figure 4:**
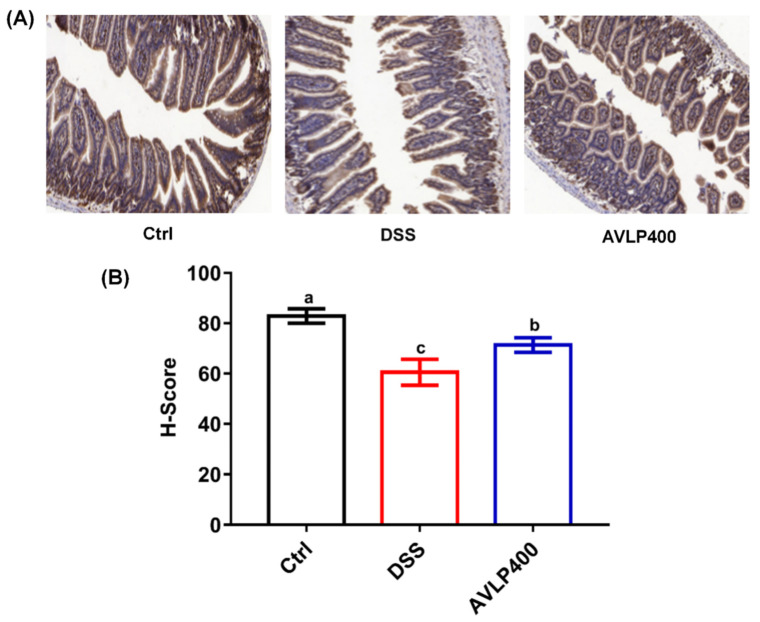
Effect of AVLP on ZO-1 protein expression in colitis mice. (**A**) Representative images of IHC staining. (**B**) H-Score of IHC staining. Significant differences (*p* < 0.05) are indicated with different letters.

Accumulating data suggest that intestinal epithelial barrier dysfunction is also an essential pathological feature of ulcerative colitis [39]. ZO-1 protein, concentrated in the intestinal epithelium, has a crucial role in sustaining integral intestinal barrier function and adjusting gut mucosal permeability [40]. Research has suggested that TNF*-α* could impair the mechanical barrier of the intestinal mucosa by adjusting ZO-1 expression [41]. In the current study, the ZO-1 protein expression was detected via immunohistochemistry (IHC). As displayed in Figure 4A,B, ZO-1 expression in colon tissue of the DSS group was significantly reduced in comparison to that in the Ctrl group (*p* < 0.05), and the H-Score dropped by approximately 33.81%. However, AVLP400 administration statistically upregulated ZO-1 expression in the colon tissue of colitis mice (*p* < 0.05), and the H-Score increased by about 22.97%. ZO-1 expression was significantly upregulated in IBD mice after cure [42]. Therefore, protecting intestinal barrier function by upregulating ZO-1 protein expression is one of the reasons why AVLP improves colitis.

### 3.5. Effect of AVLP on Gut Flora in Colitis Mice

**Figure 5:**
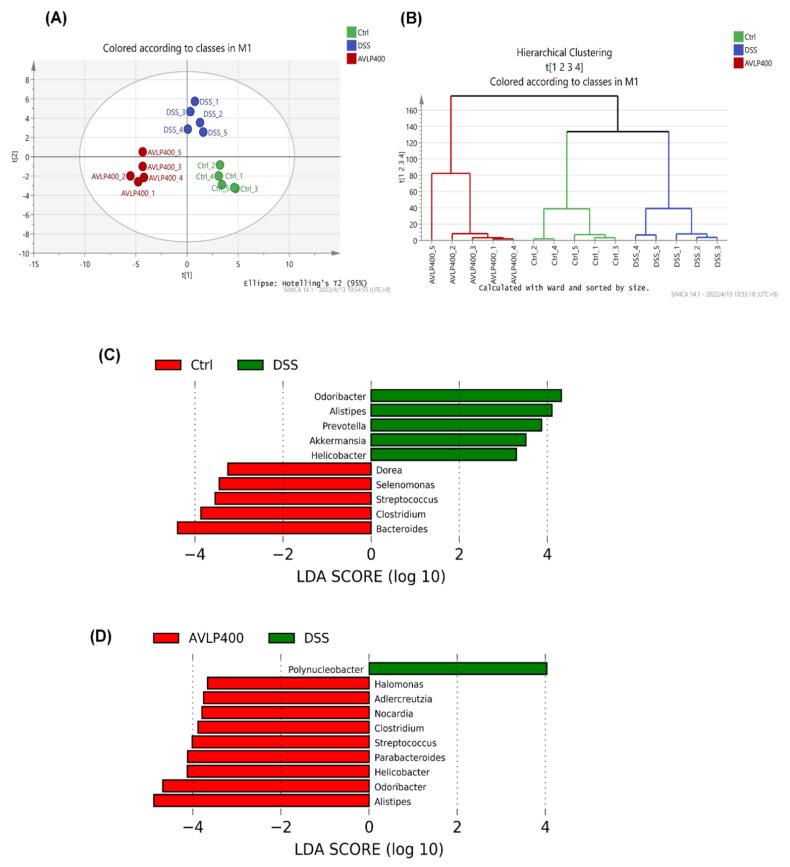
Effect of AVLP on gut flora in colitis mice. (**A**) Partial least squares discrimination analysis. (**B**) Hierarchical clustering tree analysis. (**C**) LEfSe comparison between the Ctrl and DSS groups. (**D**) LEfSe comparison between the DSS and AVLP400 groups.

Interactions between the gut microbiome and host health are gaining increasing attention. A large amount of evidence has confirmed that changes in the composition and abundance of intestinal microorganisms are strongly correlated with the health of living things [43]. The occurrence and deterioration of many diseases, such as inflammatory bowel disease and hepatic steatosis, are closely related to turbulence in intestinal flora [44]. For instance, lipopolysaccharides (LPS) that originate from the cell envelop of some bacteria might trigger an inflammatory reaction, causing intestinal disorders [45]. On the other hand, enteric microorganisms could produce a class of healthy metabolites, such as short chain fatty acids (SCFAs), by means of fermenting some compounds that are not easily digested by digestive enzymes [46]. SCFAs are the main energy sources of intestinal epithelial cells, which could accelerate cell proliferation and enhance the mechanical strength of intestinal mucosa [47]. Previous studies have adequately demonstrated that SCFA production capacity is decreased in the intestinal tracts of colitis patients and DSS-induced colitis animals [48]. Therefore, adjustments to gut microorganisms might alleviate colitis through influencing their metabolic capacity. Previously, our research results confirmed that AVLP displays antidigestion properties. Meanwhile, AVLP also possesses strong inhibitory potential for carbohydrate enzymes, suggesting that AVLP may possess the potential to regulate intestinal flora. In order to assess the effects of AVLP on the intestinal bacterial composition in mice, high-throughput sequencing (V3–V4) was executed in this study. As displayed in Figure 5A,B, the overall compositions of gut bacteria at the OTU level among the Ctrl group, DSS group, and AVLP400 group were significantly isolated, suggesting that DSS and AVLP intervention had a certain effect on the composition and abundance of gut bacteria.

At the phylum level, the *Firmicutes* abundance in the AVLP400 group was decreased by approximately 66.22% and *Bacteroidetes* was increased by 217.39% as compared with the DSS group. The *Bacteroidota*/*Firmicutes* (B/F) ratio is an efficient index to judge the change in bacterial flora, and a decreased B/F ratio is considered to be a pathological indicator of colitis [49]. In contrast to the DSS group, the AVLP400 group showed a trend of increase in the B/F ratio (*p* < 0.05), indicating that AVLP was beneficial in restoring intestinal flora in mice with DSS-induced IBD.

LEfSe comparison analysis was further carried out to investigate the difference in gut bacterium abundance at the genus level (Figure 5C,D). As shown in Figure 5C, in comparison to the Ctrl group, some bacterial genera, including *Dorea*, *Selenomonas*, *Streptococcus*, *Clostridium,* and *Bacteroides*, were substantially reduced, and the amounts of *Odoribacter*, *Alistipes*, *Prevotella*, *Akkermansia,* and *Helicobacter* were increased significantly in the DSS group (*p* < 0.05, LDA score > 2). Figure 5D shows the regulatory effects of AVLP on the gut microflora of colitis mice: AVLP administration significantly increased the abundance of *Halomonas*, *Adlercreutzia*, *Nocardia*, *Clostridium*, *Streptococcus*, *Parabacteroides*, *Helicobacter*, *Odoribacter,* and *Alistipes* and reduced the amount of *Polynucleobacter* in colitis mice. An obvious decrease in *Streptococcus* and increase in *Prevotella* are found in colitis patients [50]. Dziarski found that *Alistipes finegoldii* is a colitis-protective species and *Prevotella falsenii* is a colitis-promoting species [51]. The *Prevotella* genus was certified as a larvaceous marker associated with Crohn’s disease [52]. The above results show that AVLP possesses the potential to regulate the intestinal bacteria of colitis patients, which also provides a new direction for the industrial utilization of AVLP.

**Figure 6:**
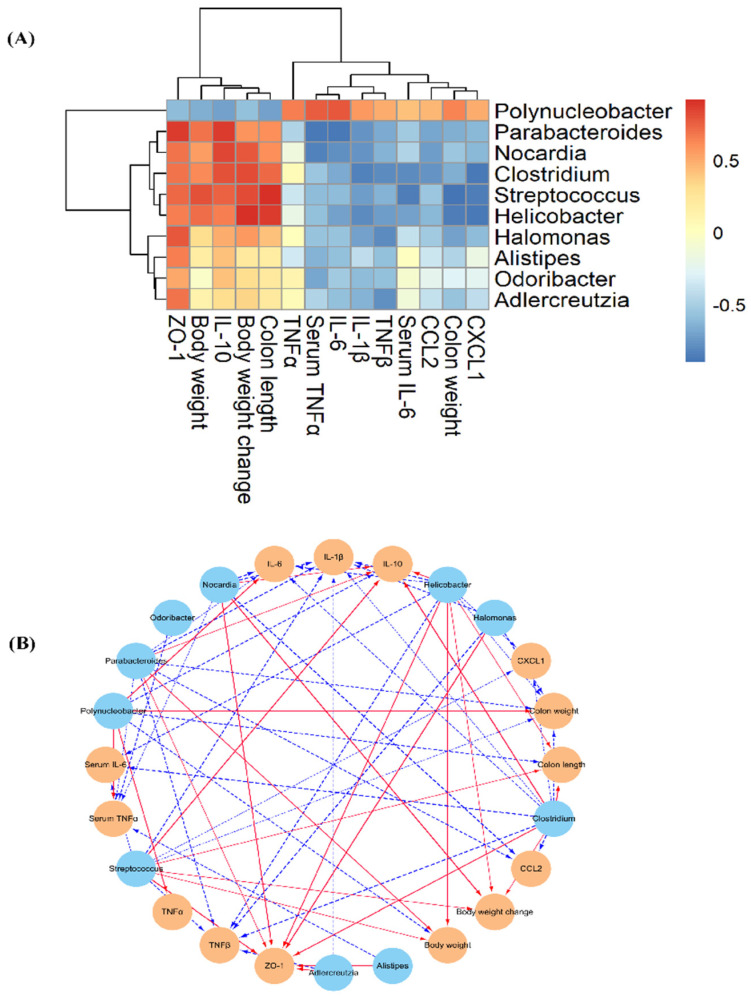
Spearman’s correlation between gut flora abundance and the improvement in colitis. (**A**) Heat map of Spearman’s correlation; (**B**) visualization of the correlation network. Note: the significant edges were drawn in the network using the Spearman correlation test (|r| > 0.6, FDR adjusted *p* < 0.05).

Furthermore, the correlation between gut bacteria and the parameters of colitis was evaluated by Spearman’s correlation analysis (Figure 6A,B). The analysis results showed that the abundances of *Streptococcus*, *Parabacteroides*, *Nocardia*, *Helicobacter,* and *Clostridium* were negatively linked with the indicators of colon weight, serum IL-6, serum TNF*α*, IL-6, CCL2, CXCL1, and TNF*β* and were positively linked with body weight, colon length, IL-10, and ZO-1. A significant negative correlation was found between the relative amounts of *Odoribacter*, *Nocardia*, *Halomonas*, *Alistipes,* and *Adlercreutzia* and the mRNA relative expressions of IL-1*β*, IL-6, and TNF*β*. In addition, *Polynucleobacter,* with higher abundance in the DSS group, was negatively correlated with body weight, colon length, IL-10, and ZO-1 and positively linked with colon weight, serum TNF*α*, IL-1*β*, IL-6, TNF*α,* and CXCL1. In line with the current research, a previous study found that bacterial genera, including *Clostridium*, *Parabacteroides*, *Adlercreutzia*, *Odoribacter,* and *Alistipes*, possess potentially beneficial effects on the improvement of inflammatory disease by enhancing the levels of SCFAs and IL-10, adjusting the balance of T cells and Th1/Th17 cells, blocking proinflammatory cytokine production, and maintaining normal intestinal barrier function [53,54]. *Polynucleobacter* is involved in aspartate synthesis, which acts as an alternative carbon source for glutamine to power diseased cells, illustrating that AVLP normalizes intestinal glutamine in colitis mice [55]. Regrettably, due to the limitations of 16S sequencing, we did not acquire more information on the effects of AVLP on the gut flora at the species level. In our future research, we will further assess the regulating effect of AVLP on the gut flora at the species level by metagenomic sequencing, and we will verify the latent relationship between the regulating effect of gut flora and the anticolitis activity of AVLP through flora transplantation experiments.

## 4. Conclusions

In summary, AVLP administration effectively alleviated the symptoms of DSS induced-colitis in a mouse model, which may be attributed to its anti-inflammatory activity, improving intestinal barrier function and regulating intestinal flora. The current research suggests that AVLP supplementation could be a promising candidate medicine and drug adjuvant for inflammatory bowel disease in humans.

## Figures and Tables

**Table 1:** Primer sequences for real-time PCR.

Gene	Forward Primer	Reverse Primer
Il-1*β*	ATGCCACCTTTTGACAGTGATG	GATGTGCTGCTGCGAGATTT
IL-6	GACTTCCATCCAGTTGCCTT	ACAACTCTTTTCTCATTTCCACGA
IL-10	CTTACTGACTGGCATGAGGATCA	GCAGCTCTAGGAGCATGTGG
TNF*α*	AGCCGATGGGTTGTACCTTG	ATAGCAAATCGGCTGACGGT
TNF*β*	GGAGGCATGTTCGGTAGTGG	CCCTGCGTTGGATTTCGTG
CCL2	CCAGCAAGATGATCCCAATGAGT	CCATTCCTTCTTGGGGTCAGC
CXCL-1	ACCCAAACCGAAGTCATAGCC	ACTTGGGGACACCTTTTAGCATC
GAPDH	TGTTTCCTCGTCCCGTAGACA	AACAATCTCCACTTTGCCACT

## Data Availability

Not applicable.
